# Supplementary figures and images for: Insights into substrate recognition and specificity for IgG by Endoglycosidase S2
Source: PLoS Comput Biol. 2021 Jul 26;17(7):e1009103. doi: 10.1371/journal.pcbi.1009103 (PMC8354483; doi:10.1371/journal.pcbi.1009103)

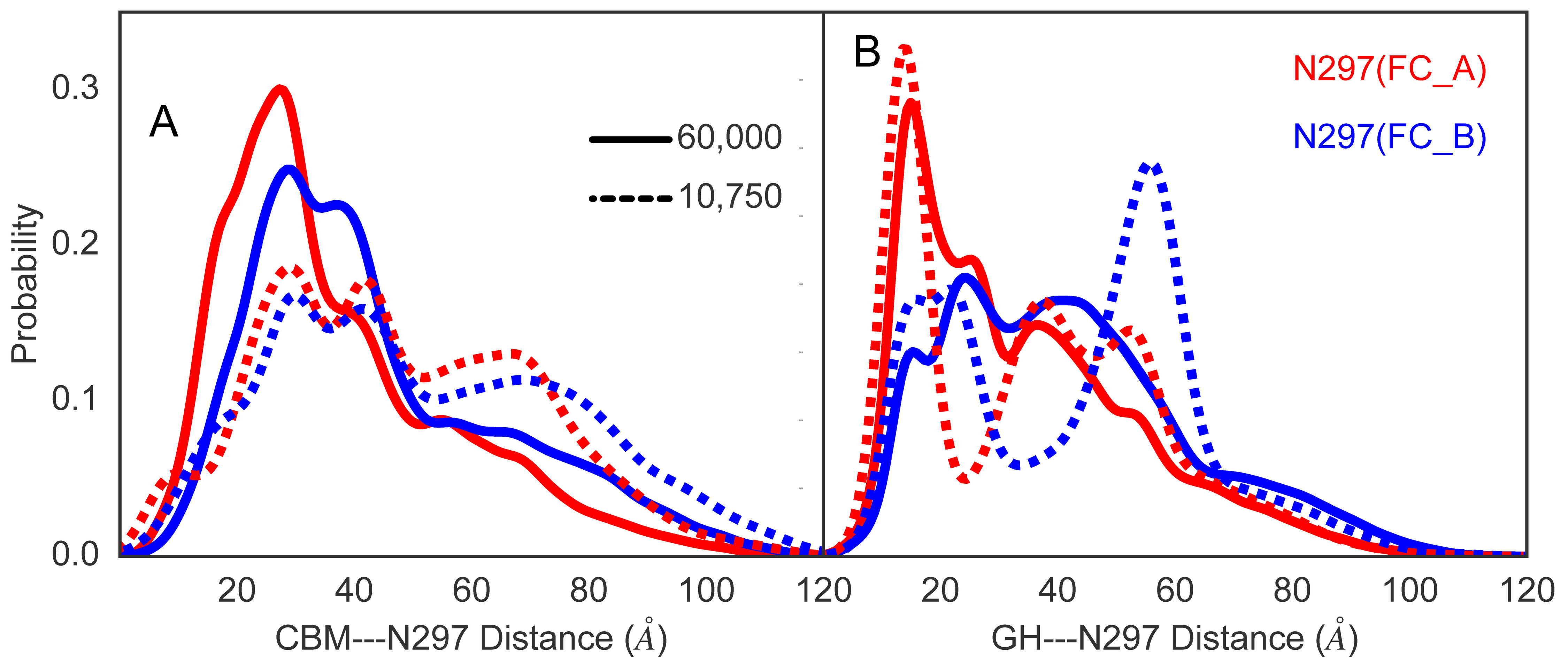

Supplement: S1 Fig — Distribution of distances between N297 and the center-of-mass of A) CBM glycan binding site or B) GH active site. Red and blue lines correspond to N297 from Fc chain A and B, respectively. Distributions of initial 60,000 complex structures are represented with solid lines and those of the 10,750 structures remaining after the first center-of-mass filter are represented by dotted lines. (TIF) [file pcbi.1009103.s001.tif]

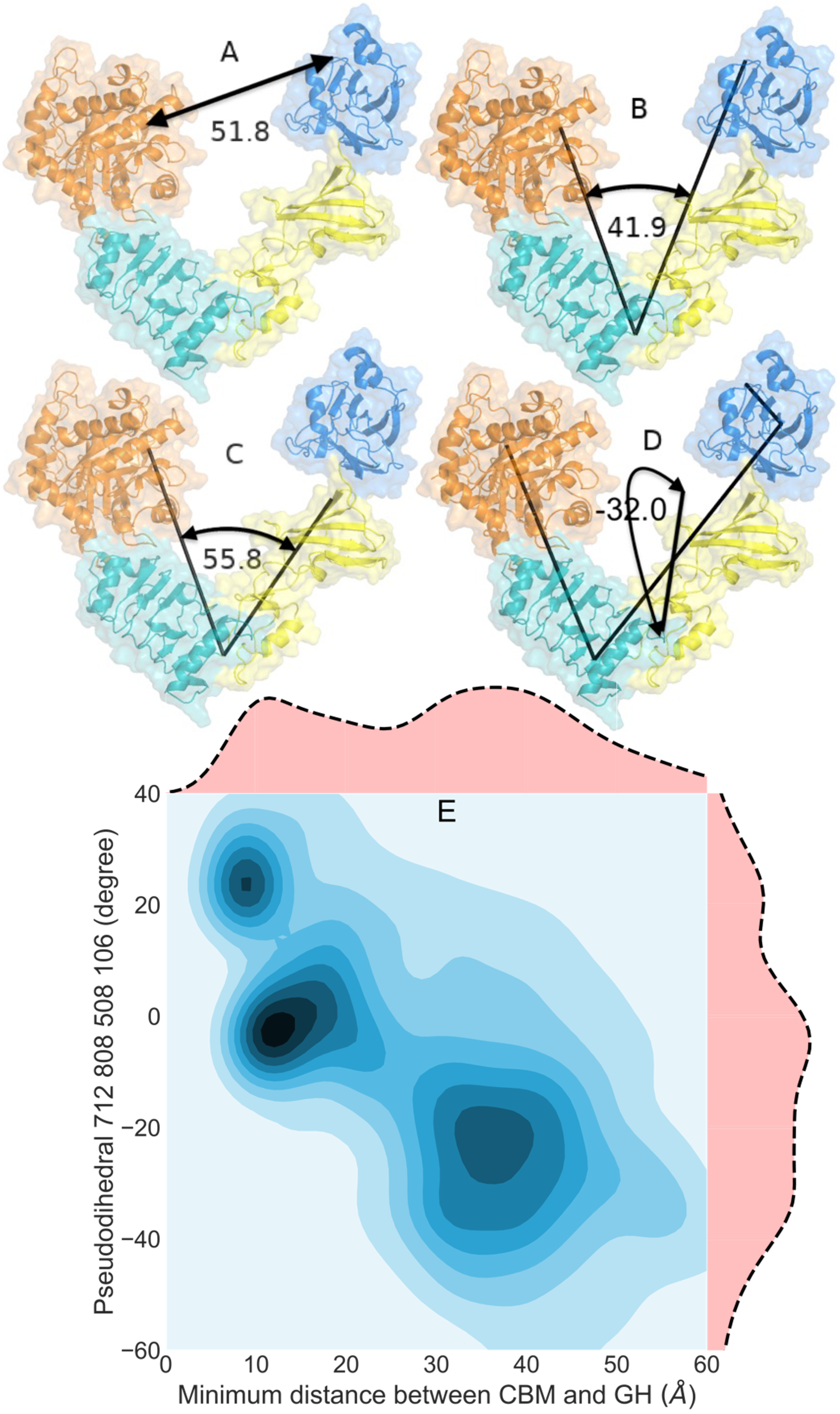

Supplement: S2 Fig — Annotation of distance, angles and pseudodihedral angle defining the location of the CBM relative to the GH domain (A-D) and (E) a 2-dimensional plot of the minimum distance between the CBM and GH (A) and the pseudodihedral angle (panel D) combined from the 4 apo-EndoS2 simulations (4 simulations for a total of 8 μs). The (A) distance was based on the minimum distance between the CBM and GH non-hydrogen atoms, angle B was based on the Ca atoms of residues 712 (on CBM), 548 (on LLR) and 186 (on GH), angle C was based on Ca atoms of residues 639 (on hybrid-Ig domain), 548 (on LLR) and 186 (on GH), and the pseudodihedral angle D was defined by the Ca atoms of residue 712 (on CBM), 808 (on CBM), 508 (on LLR) and 106 (on GH). As is evident sampling of shorter distances between the CBM and GH in the closed state is associated with pseudodihedral angles approaching 0°, while the larger distances associated with the open state correspond to larger pseudodihedral angles in the range of 30 to 50°. This shows that conformational difference between the open and closed states of EndoS2 have significant contributions from rotation of the CBM around the hinge between the CBM and hybrid-Ig. (TIF) [file pcbi.1009103.s002.tif]

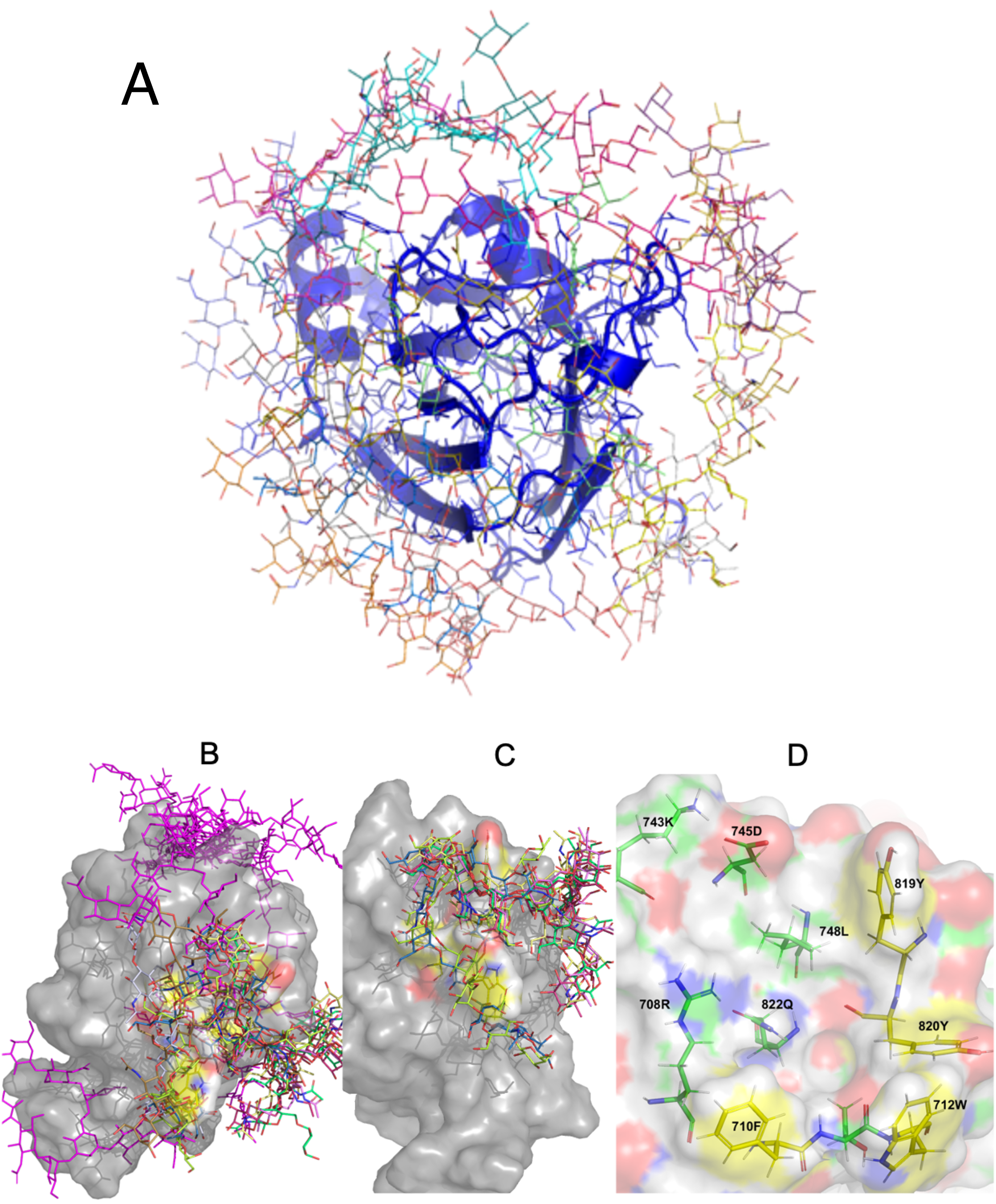

Supplement: S3 Fig — A. Aggregated initial orientations of the CT glycan surrounding the CBM. Blue cartoon represents the CBM protein and the glycans are shown as sticks in the initial locations approximately 10 Å from the protein surface used to initiate the SILCS-MC calculations from which a putative glycan binding site on the CBM was identified as described in the computational methods. B-D. CBM binding pocket identified based on experimental data and SILCS-MC docking of glycans with the CBM. B) Top 15 and C) top 6 SILCS-MC docked conformations based on the LGFE scores, and D) amino acids identified based on experimental data [25] (yellow carbons) and those identified based on the 6 lowest LGFE SILCS conformations and hydrogen bond analysis (green carbons). (TIF) [file pcbi.1009103.s003.tif]

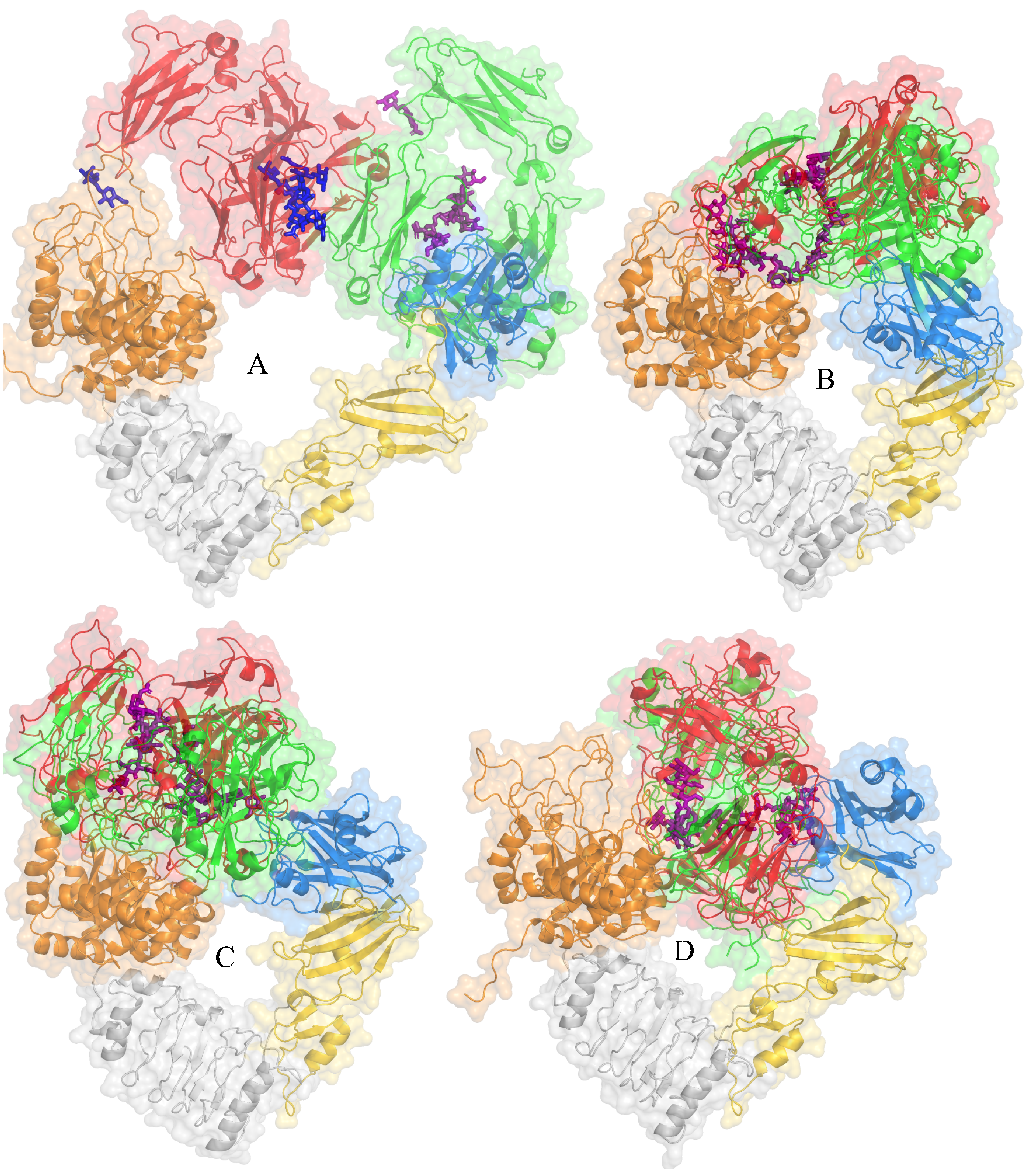

Supplement: S4 Fig — Shift of the Fc on EndoS2 from the selected MD simulations of model A, model B, model C and model D. White cartoon represents the EndoS2 with the CBM (blue) and the GH (orange) based on the initial simulation 2 coordinates used to start the complex simulation (except model C where simulation 3 is presented). The initial orientation of the Fc in the models used to initiate the MD simulation are shown in green cartoon and the orientation of the Fc from the 2 μs time frame is shown in red cartoon following alignment of EndoS2 to the initial coordinates excluding CBM in the alignment due to its rotation during the MD simulation. Glycans in the initial orientation are shown as purple sticks as in the final orientation as blue sticks. For visualization EndoS2 was removed from the final frame. (TIF) [file pcbi.1009103.s004.tif]

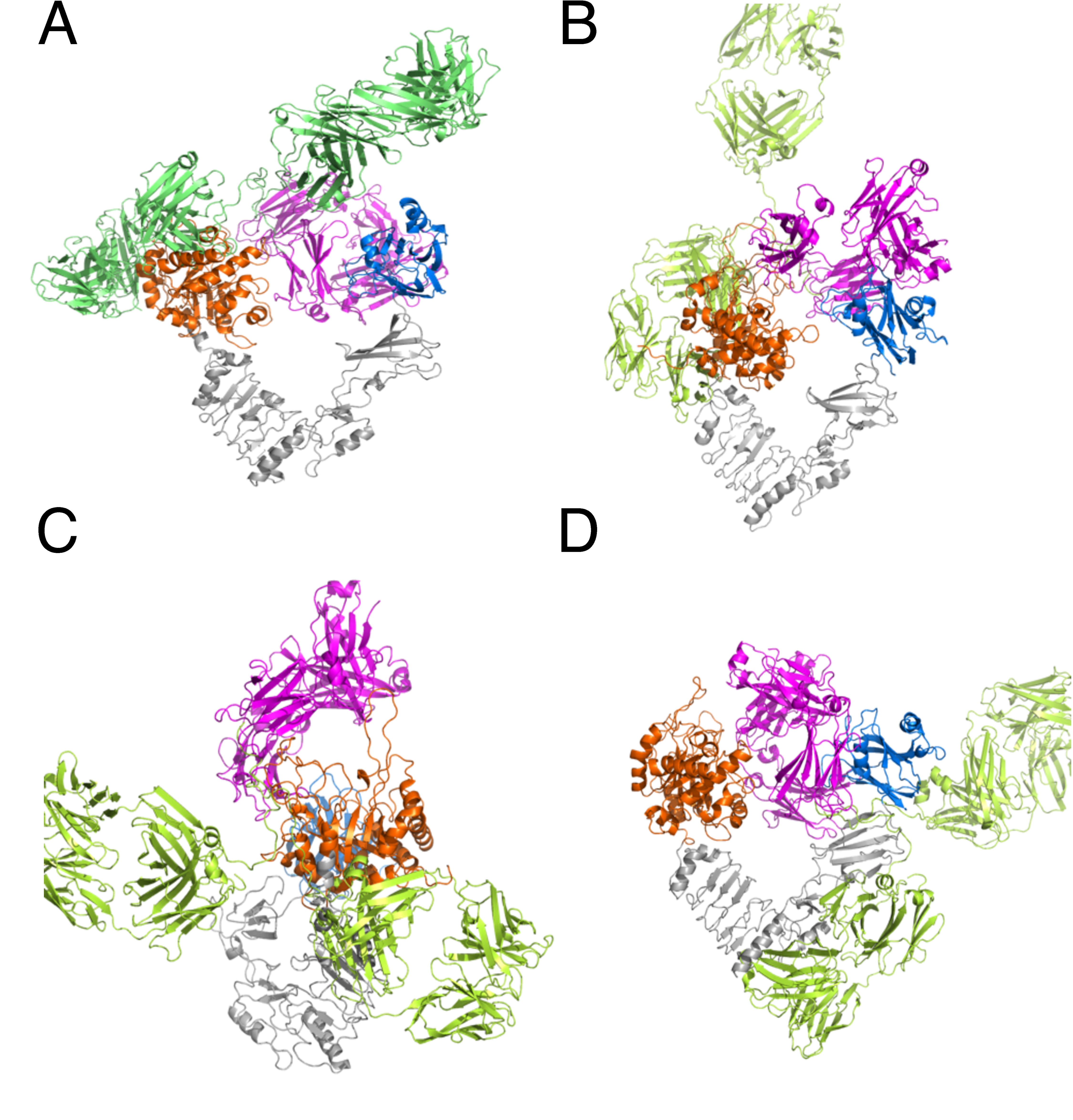

Supplement: S5 Fig — A. Full Antibody-EndoS2 complex based on model A. The mAb structure is based on PDB 1IGT [31] following RMSD alignment of the nonhydrogen atoms in the Fc from the model A simulation. The Fc is pink, Fabs are green, GH is orange, CBM is blue and the remainder of EndoS2 is gray. The image indicates that the model A structure can accommodate the full antibody structure given the flexibility of the linker between the Fc and Fabs. B. Full Antibody-EndoS2 complex based on model B. The mAb structure is based on PDB 1IGT [31] following RMSD alignment of the nonhydrogen atoms in the Fc from the model B simulation. The Fc is pink, Fabs are green, GH is orange, CBM is blue and the remainder of EndoS2 is gray. The image indicates that the model B structure can accommodate the full antibody structure given the flexibility of the linker between the Fc and Fabs. C. Full Antibody-EndoS2 complex based on model C. The mAb structure is based on PDB 1IGT [31] following RMSD alignment of the nonhydrogen atoms in the Fc from the model C simulation. The Fc is pink, Fabs are green, GH is orange, CBM is blue and the remainder of EndoS2 is gray. The image indicates that the model C structure can accommodate the full antibody structure given the flexibility of the linker between the Fc and Fabs. D. Full Antibody-EndoS2 complex based on model D. The mAb structure is based on PDB 1IGT [31] following RMSD alignment of the nonhydrogen atoms in the Fc from the model D simulation. The Fc is pink, Fabs are green, GH is orange, CBM is blue and the remainder of EndoS2 is gray. The image indicates that the model D structure can accommodate the full antibody structure given the flexibility of the linker between the Fc and Fabs. (TIF) [file pcbi.1009103.s005.tif]

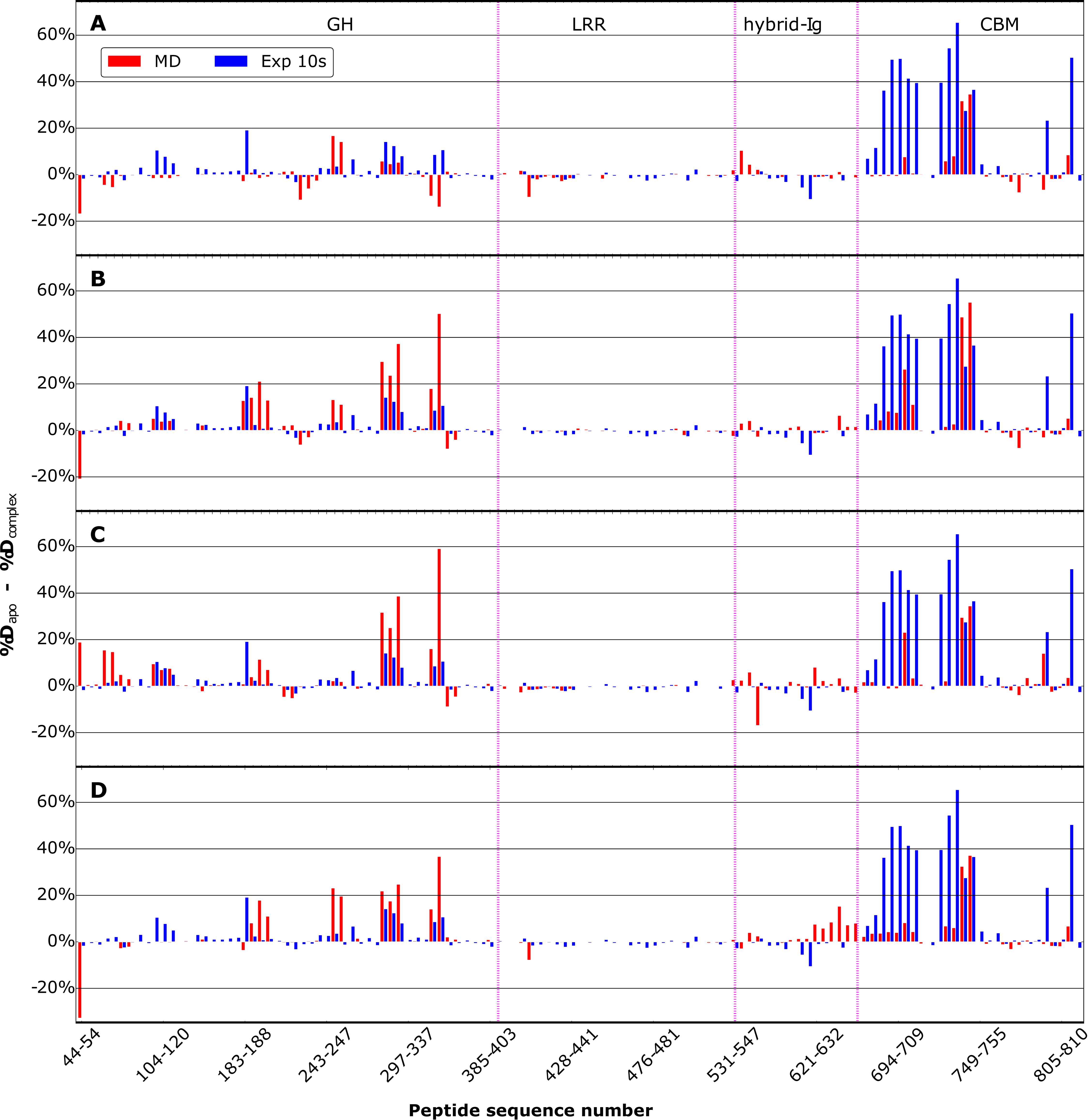

Supplement: S6 Fig — Simulation vs experimental hydrogen-deuterium exchange percent differences for A) model A, B) model B, C) model C and D) model D for the diglycosylated species only for all four models. Differences in the experimental percent deuteration for peptides from EndoS2 in the unliganded and IgG1-complexed states [25] over the first 10s are plotted as blue bars. The analogous differences in the MD calculated percent deuteration for peptides from the EndoS2-apo simulations and from the Fc-glycan-EndoS2 model complex states as red bars. The different domains of Endos2 are labelled across the top of the figure and separated by purple dashed lines as follows: Glycoside hydrolase (GH), leucine-rich repeat (LRR), hybrid-Ig, and carbohydrate-binding module (CBM). Individual peptides are plotted on the X-axis from the N- to C-terminus based on the sequence number of the first residue in the peptide. (TIF) [file pcbi.1009103.s006.tif]

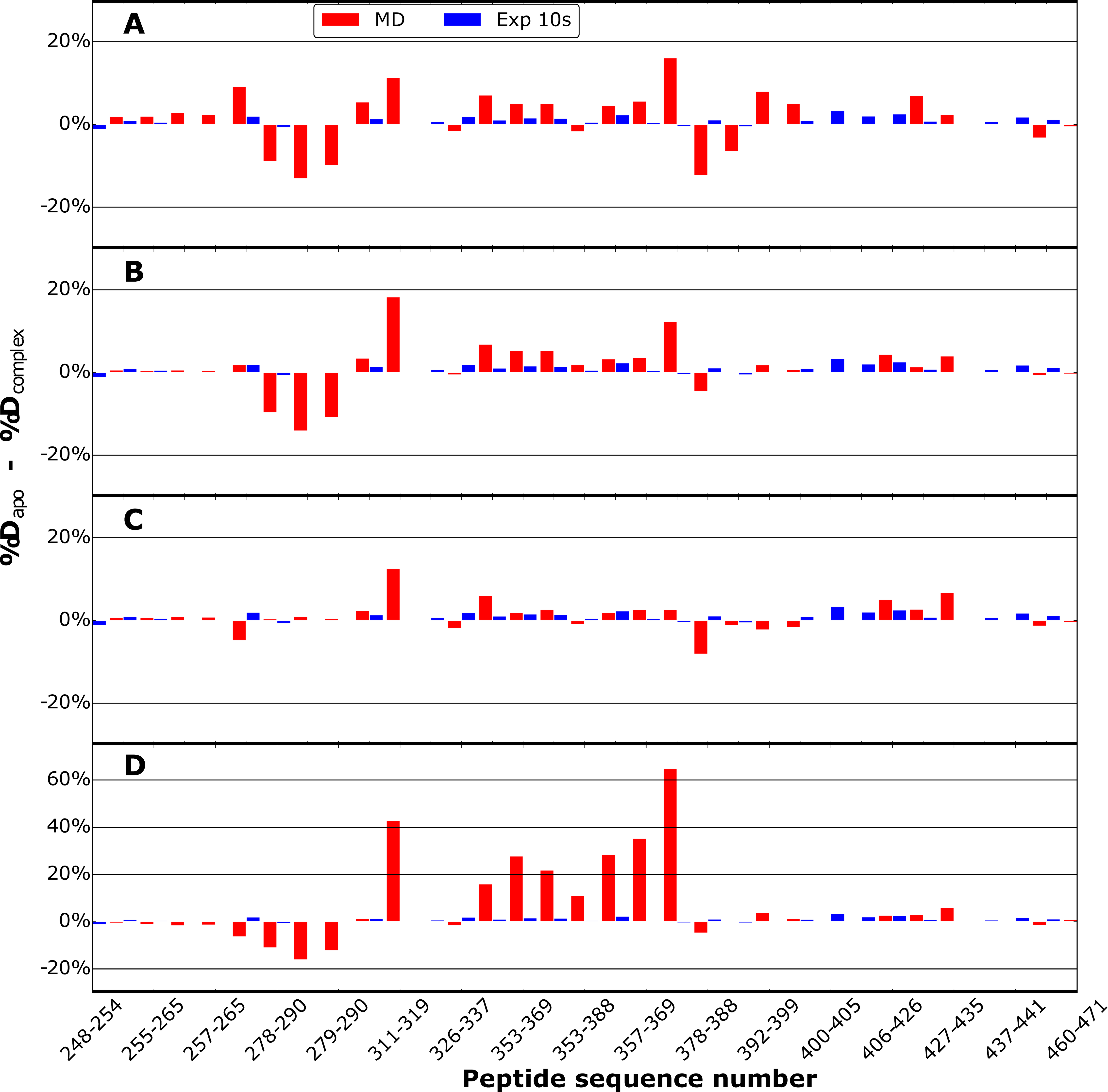

Supplement: S7 Fig — Simulation vs experimental hydrogen-deuterium exchange for A) model A, B) model B, C) model C and D) model D for the diglycosylated species only for all four models. Differences in the experimental percent deuteration for peptides from Fc in the unliganded and IgG1-complexed states [25] over the first 10s are plotted as blue bars. The analogous differences in the MD calculated percent deuteration for peptides from the Fc apo simulations and from the Fc-glycan-EndoS2 model complex states as red bars. Individual peptides are plotted on the X-axis from the N- to C-terminus based on the sequence number of the first residue in the peptide. Note the change in the Y-axis in panel D versus panels A, B and C. (TIF) [file pcbi.1009103.s007.tif]

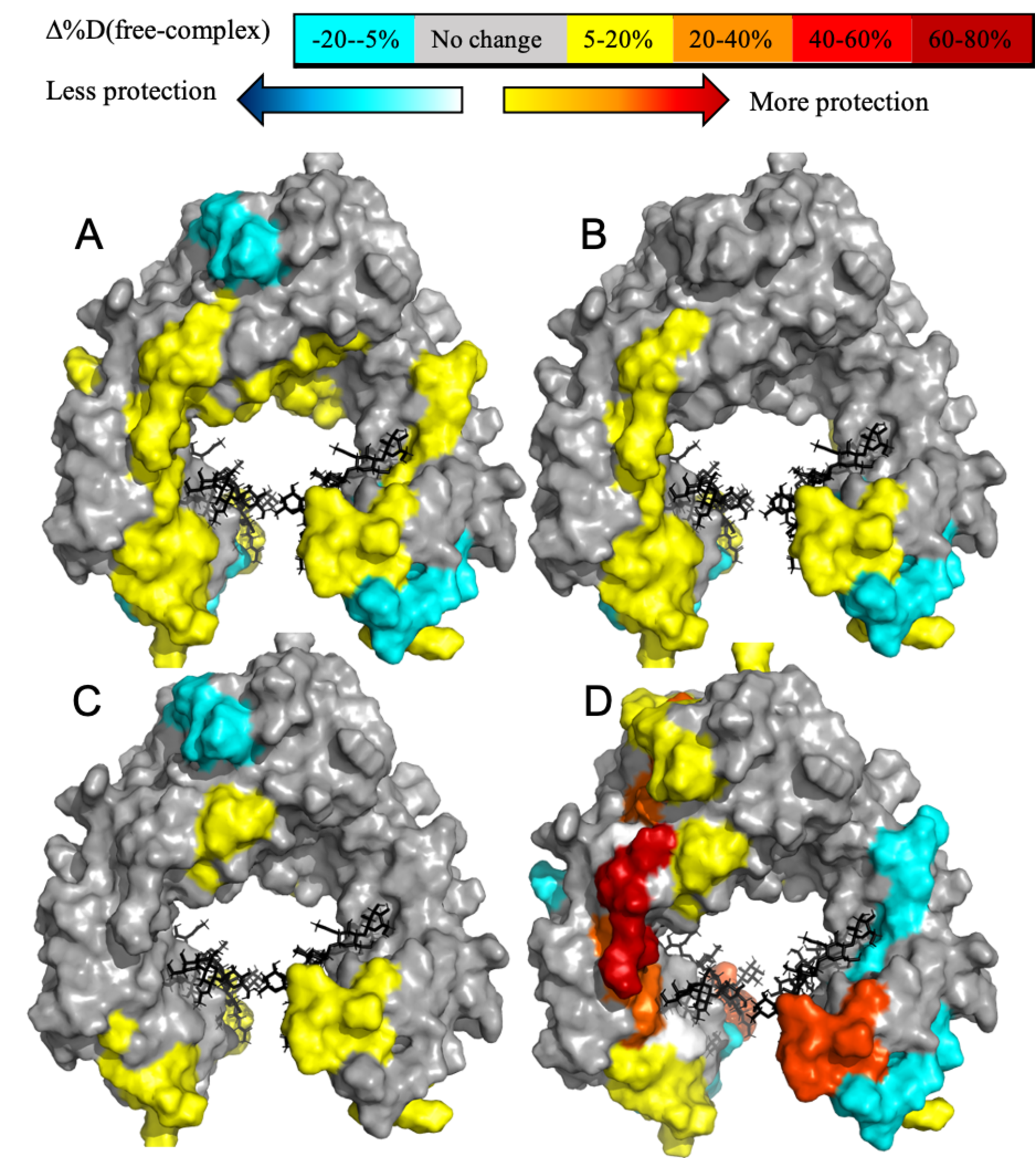

Supplement: S8 Fig — [31] The predicted peptide hydrogen-deuterium exchange is average of diglycosylated Fc for A) model A, B) model B, C) model C and D) model D. (TIF) [file pcbi.1009103.s008.tif]
