# Supplementary material for: Insights into substrate recognition and specificity for IgG by Endoglycosidase S2
Source: PLoS Comput Biol. 2021 Jul 26;17(7):e1009103. doi: 10.1371/journal.pcbi.1009103 (PMC8354483; doi:10.1371/journal.pcbi.1009103)
Supplement: S1 Table — (DOCX) [file pcbi.1009103.s009.docx]

S1 Table. Summary of the simulation systems and simulation times used to generate conformations for EndoS2-Fc-glycan model reconstruction and final EndoS2-Fc-glycan complex.

| System Name | Simulation Time | Simulation Type | Simulation package | Box Size | # Total Atoms |
| --- | --- | --- | --- | --- | --- |
| Mono-glycosylated FC | 250 ns | HREST-bpCMAP | CHARMM-OpenMM[[52](#_ENREF_52)] | 97Åx97Åx97Å | 91934 |
| Di-glycosylated FC | 250 ns | HREST-bpCMAP | CHARMM-OpenMM[[52](#_ENREF_52)] | 97Åx97Åx97Å | 93260 |
| EndoS2 | 4x2 μs = 8 μs | Standard MD | OpenMM[[46](#_ENREF_46)] | 132Åx132Åx132Å | 216540 |
| Model A | Run1 (3.5 μs) | Standard MD | OpenMM[[46](#_ENREF_46)] | 156Åx156Åx156Å | 357993 |
|  | Run2 (3.5 μs) |  |  | 166Åx166Åx166Å | 430774 |
|  | Run3 (3.5 μs) |  |  | 156Åx156Åx156Å | 351507 |
|  | Run4 (5.2 μs) |  |  | 155Åx155Åx155Å | 363182 |
| Model B | Run1 (3 μs) | Standard MD | OpenMM[[46](#_ENREF_46)] | 158Åx158Åx158Å | 373267 |
|  | Run2 (3 μs) |  |  | 157Åx157Åx157Å | 363122 |
|  | Run3 (3 μs) |  |  | 131Åx131Åx131Å | 211018 |
|  | Run4 (3 μs) |  |  | 141Åx141Åx141Å | 292264 |
| Model C | Run1 (3 μs) | Standard MD | OpenMM[[46](#_ENREF_46)] | 156Åx156Åx156Å | 357885 |
|  | Run2 (5.5 μs) |  |  | 155Åx155Åx155Å | 351780 |
|  | Run3 (3 μs) |  |  | 154Åx154Åx154Å | 343672 |
|  | Run4 (3 μs) |  |  | 164Åx164Åx164Å | 414424 |
| Model D | Run1 (2 μs) | Standard MD | OpenMM[[46](#_ENREF_46)] | 139Åx139Åx139Å | 253096 |
|  | Run2 (2 μs) |  |  | 131Åx131Åx131Å | 210763 |
|  | Run3 (2 μs) |  |  | 142Åx142Åx142Å | 268197 |
|  | Run4 (2 μs) |  |  | 132Åx132Åx132Å | 215561 |
