# Supplementary material for: Insights into substrate recognition and specificity for IgG by Endoglycosidase S2
Source: PLoS Comput Biol. 2021 Jul 26;17(7):e1009103. doi: 10.1371/journal.pcbi.1009103 (PMC8354483; doi:10.1371/journal.pcbi.1009103)
Supplement: S2 Table — Restraints were only applied to non-hydrogen atoms and units are kcal/mol/Å2. (DOCX) [file pcbi.1009103.s010.docx]

S2 Table. Harmonic restraints applied during the equilibration MD simulations of the full EndoS2-Fc-glycan model complexes in aqueous solution. Restraints were only applied to non-hydrogen atoms and units are kcal/mol/Å^2^.

| Simulation time | MD Time Step | Carbohydrate | Backbone | Sidechain | Loop backbone | Loop side chain |
| --- | --- | --- | --- | --- | --- | --- |
| 0.5 ns | 1 fs | 40.0 | 400.0 | 200.0 | 50.0 | 10.0 |
| 2 ns | 2 fs | 4.0 | 40.0 | 10.0 | 5.0 | 1.0 |
| 2 ns | 2 fs | 0.0 | 1.0 | 0.0 | 0.0 | 0.0 |
