# Supplementary material for: Insights into substrate recognition and specificity for IgG by Endoglycosidase S2
Source: PLoS Comput Biol. 2021 Jul 26;17(7):e1009103. doi: 10.1371/journal.pcbi.1009103 (PMC8354483; doi:10.1371/journal.pcbi.1009103)
Supplement: S1 Text — (PDF) [file pcbi.1009103.s011.pdf]

Supporting Information

Insights into substrate recognition and specificity for IgG by Endoglycosidase S2.

Short title: Molecular modeling and dynamics simulations of Endoglycosidase S2-Fc complexes.

Asaminew H. Aytenfisu[1], Daniel Deredge[1], Erik H. Klontz[2], Jonathan Du[2,3], Eric J. Sundberg[2,3], Alexander D. MacKerell Jr. [1]*

[1]University of Maryland Computer-Aided Drug Design Center, Department of Pharmaceutical Sciences, School of Pharmacy, University of Maryland, Baltimore, Maryland 21201, United States

[2]Department of Medicine, University of Maryland School of Medicine, Baltimore, Maryland 21201, United States

[3]Department of Biochemistry, Emory University School of Medicine, Atlanta, GA 30322, USA

*Corresponding Author

E-mail: alex@outerbanks.umaryland.edu; Telephone: +1-410-706-7442.

Workflow 1: CHARMM code for loop reconstruction

The following lines were used in the CHARMM input.

```
Add loop definition here
set e0 ?ener                              ! system energy
set b 1
label nextloop
block 2
  call 2 sele .not. loop@b  end           ! block with loop deleted
  coeff 1 1 0.0
  coeff 1 2 0.0
  coeff 2 2 1.0                           ! self energy with loop excluded
end
energy
block
   clear
end
if ?ener .le. @e0 then                    ! check if energy improves
set loop_id @b                            ! save loop id
set e0 ?ener                              ! update energy
endif
incr b by 1
if @b .le. 8 goto nextloop                ! loop over all 8 loops

! after identifying the problematic loop, next is read through the loop library and rebuild
energy                                    ! system energy
set e0 ?ener
set i 1
label nextframe
open read unit 10 card name ../crd-loop/loop@loop_id.@i.crd  ! read loop coordinate
read coor unit 10 card resid
coor orient rms mass sele lpr@loop_id end          ! reorient with respect to the loop
open read unit 10 card name final/@crdnam.crd
read coor unit 10 card resid sele .not. loop@loop_id end       ! replace the rest of the complex
back
energy                                    ! evaluate energy
if ?ener .le. @e0 then                    ! check if the new coordinate is better
set d @i                                  ! save trajectory frame number
set e0 ?ener
endif

incr i by 1
if @i .le. 100 goto nextframe
```
